# PPARγ signaling is required for mediating EETs protective effects in neonatal cardiomyocytes exposed to LPS

**DOI:** 10.3389/fphar.2014.00242

**Published:** 2014-11-11

**Authors:** Victor Samokhvalov, Jelle Vriend, Kristi L. Jamieson, Maria K. Akhnokh, Rajkumar Manne, John R. Falck, John M. Seubert

**Affiliations:** ^1^Faculty of Pharmacy and Pharmaceutical Sciences, University of AlbertaEdmonton, AB, Canada; ^2^Department of Chemistry and Pharmaceutical Sciences, Faculty of Sciences, VU UniversityAmsterdam, Netherlands; ^3^Department of Biochemistry and Pharmacology, University of Texas Southwestern Medical CenterDallas, TX, USA; ^4^Department of Pharmacology, Faculty of Medicine and Dentistry, University of AlbertaEdmonton, AB, Canada

**Keywords:** epoxyeicosatrienoic acid, cardiac cells, LPS, inflammation, PPARγ

## Abstract

Lipopolysaccharide (LPS) is a bacterial wall endotoxin producing many pathophysiological conditions including myocardial inflammation leading to cardiotoxicity. Epoxyeicosatrienoic acids (EETs) are biologically active metabolites of arachidonic acids capable of activating protective cellular pathways in response to stress stimuli. EETs evoke a plethora of pathways limiting impairments of cellular structures, reducing cell death, and promoting anti-inflammatory reactions in various cell types. Considering EETs are capable of producing various biological protective effects, we hypothesized that EETs would protect rat neonatal cardiomyocytes (NCM) against LPS-induced cytotoxicity. In this study, we used a dual-acting, synthetic analog of EETs, UA-8 [13-(3-propylureido)tridec-8-enoic acid], possessing both EET-mimetic and soluble epoxide hydrolase selective inhibitory properties and 14,15-EET as a model of canonical EET molecules. We found that both UA-8 and 14,15-EET significantly improved cell viability and mitochondrial function of cardiomyocytes exposed to LPS. Furthermore, treatment with UA-8 or 14,15-EET resulted in significant attenuation of LPS-triggered pro-inflammatory response, caspase-3 activation and reduction in the total antioxidant capacity in cardiomyocytes. Importantly, EET-mediated effects were significantly reduced by pharmacological inhibition of peroxisome proliferator-activated receptors γ (PPARγ) suggesting that PPARγ signaling was required for EETs exerted protective effects. Data presented in the current study demonstrate that activation of PPARγ signaling plays a crucial role in EET-mediated protection against LPS-cytotoxicity in cardiomyocytes.

## INTRODUCTION

Inflammation is a complex and highly orchestrated process involved in protecting cells from injury; yet, mounting evidence suggests that overactivated inflammatory responses contribute to the initiation and development of a wide range of diseases including cardiovascular diseases (CVDs) (Gabay and Kushner, 1999; Ridker and Silvertown, 2008). Bacterial endotoxin lipopolysaccharide (LPS) is considered one of the major causes in initiating low-grade systemic inflammation associated with cardiac dysfunction (Niebauer et al., 1999; Opal et al., 1999). Binding of LPS to TLR-4 receptors results in execution of the IKK-NF-kB inflammatory program (Akira et al., 2001) leading to the release of pro-inflammatory cytokines such as TNFα, IL-6, IL-1, and MCP-1 (Ohlsson et al., 1990; Carlson et al., 2005; Fallach et al., 2010). The LPS-triggered release of pro-inflammatory cytokines can directly cause cardiac damage via numerous mechanisms including activation of JNK signaling (Hambleton et al., 1996; Charalambous et al., 2007; Drosatos et al., 2011), increased oxidative stress (Ben-Shaul et al., 2001), decreased β-adrenergic activity (Yasuda and Lew, 1997), reduced peroxisome proliferator-activated receptors (PPARs) expression and DNA binding activity (Feingold et al., 2004; Maitra et al., 2009; Samokhvalov et al., 2012) or direct mitochondrial damage (Choumar et al., 2011). Although LPS triggered responses are well documented, the precise cellular and molecular mechanism(s) involved in LPS-induced myocardial dysfunction remains very poorly delineated.

Arachidonic acid is a 20-carbon polyunsaturated fatty acid found in cell membranes. Activation of phospholipase A2 results in the release of arachidonic acid, which can undergo enzymatic conversion forming biologically active lipid molecules (Rosenthal et al., 1995; Roman, 2002; Levick et al., 2007). Cytochrome P450 (CYP) epoxygenases are known to metabolize arachidonic acid into four regioisomeric epoxide metabolites, epoxyeicosatrienoic acids (EETs): 5,6-, 8,9-, 11,12-, and 14,15-EET (Fang et al., 2001; Kim et al., 2004). Reported EET-mediated effects include enhanced autophagy (Samokhvalov et al., 2013), inhibition of apoptosis (Dhanasekaran et al., 2008), mitochondrial protection (Katragadda et al., 2009) and cell proliferation (Imig, 2012). Numerous *in vitro* and *in vivo* studies provide strong evidence that EETs have anti-inflammatory properties (Node et al., 1999; Deng et al., 2010; Imig, 2012), which involves inhibition of the IKK-NF-kB cascade (Rompe et al., 2010). For example, 11,12-EET was found to prevent LPS-triggered activation of the inflammatory response in monocytes by suppressing NF-kB signaling (Kozak et al., 2003). However, the exact role EETs have in regulating anti-inflammatory reactions in cardiac cells remains unknown.

Preventing a pathological activation of the inflammatory response requires a tight coordination of biological processes directed to effectively suppress the pro-inflammatory response while promoting anti-inflammatory reactions (Chinetti et al., 2001; Jones et al., 2002; Liu et al., 2005; Moraes et al., 2006). PPARs are ligand-activated transcription factors and members of the nuclear hormone receptor superfamily (Wray and Bishop-Bailey, 2008). PPAR nuclear receptors sense various biological molecules and regulate many cellular functions such as fatty acid metabolism and lipid transport (Desvergne and Wahli, 1999), inflammatory responses (Wang et al., 2002; Moraes et al., 2006), cell differentiation (Barak et al., 1999) and tissue development (Rosen et al., 1999). There are three PPAR isoforms characterized (α, γ, and β/δ) that regulate physiologically distinct processes (Delerive et al., 2001; Bocher et al., 2002). Importantly, activation of PPARs, particularly PPARγ, suppresses NF-kB-induced expression of inflammatory cytokines (Liu et al., 2005; Wang et al., 2010). Interestingly, EETs have been identified as potent PPARs activators (Node et al., 1999; Liu et al., 2005; Ng et al., 2007), suggesting that anti-inflammatory effects of EETs might be mediated via PPAR-signaling. Despite already published studies, the existing knowledge regarding the mechanisms through which EETs attenuate LPS-induced cytotoxicity appears to be insufficient. Considering LPS down-regulates PPAR-mediated signaling, thus initiating the pro-inflammatory response, our objective was to determine if the anti-inflammatory effects of EETs required activation of PPARγ signaling in neonatal cardiomyocytes (NCMs).

## MATERIALS AND METHODS

### CELL CULTURE

Neonatal cardiomyocytes were isolated from 3 day-old pups as described before (Samokhvalov et al., 2012). Each isolation was done on a different day to perform a separate set of experiments (*N* = 3–4). Isolated NCMs were cultivated in DMEM medium supplemented with 10% FBS at 37°C in a humidified incubator maintaining 5% CO_2_ and 95% air. Cell viability was assessed using a Trypan Blue exclusion assay as previously described (Samokhvalov et al., 2013). Beating rate of cardiomyocytes was evaluated by counting the number of beats per min in five different cell clusters in five independently blinded experiments.

### TREATMENT PROTOCOLS

**FIGURE 1:**
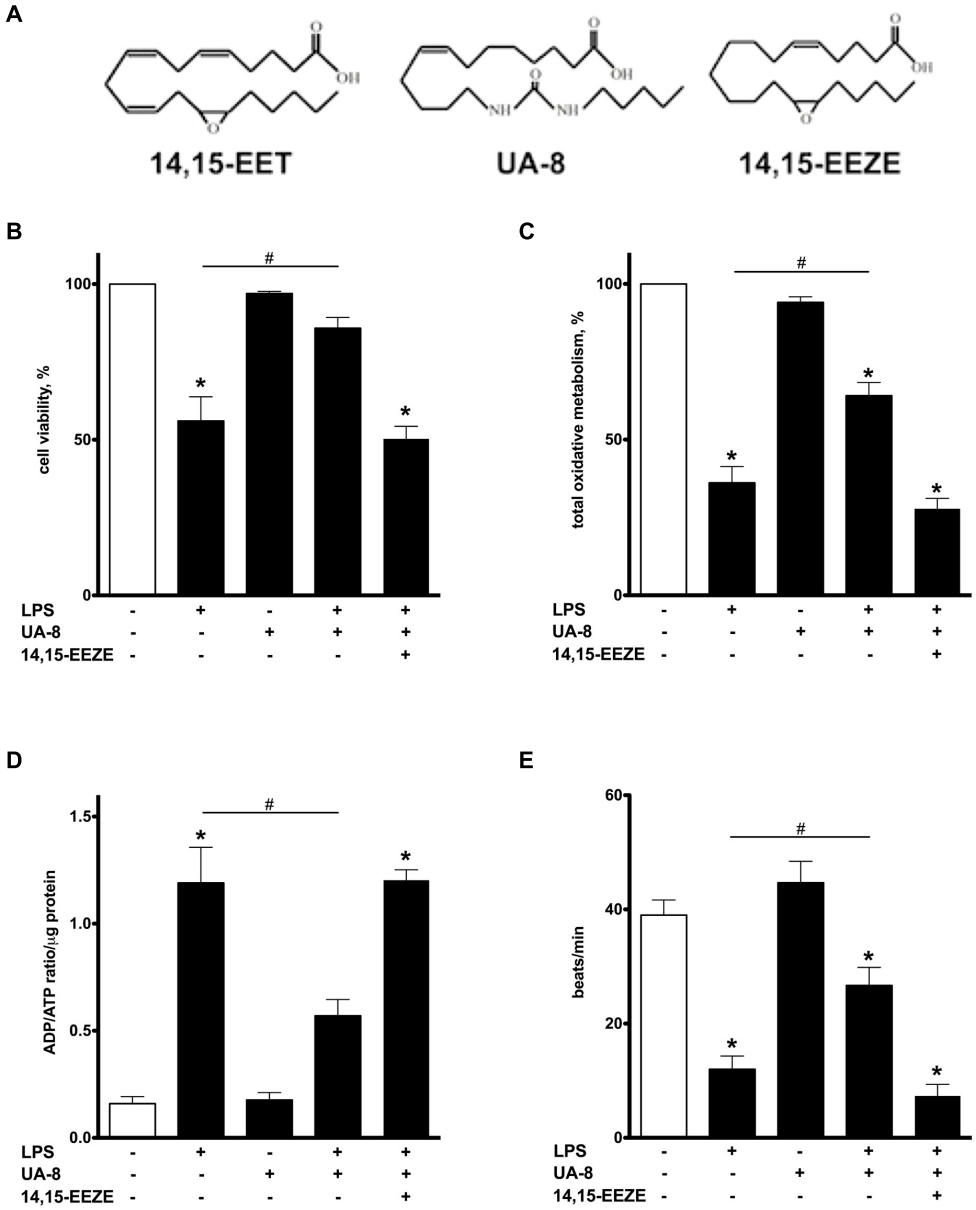
**Treatment with UA-8 attenuates LPS-induced decrease in cell viability, oxidative metabolic activity and improves contractility of neonatal cardiomyocytes.** NCMs were treated with LPS (1μg/ml) and/or UA-8 (1 μm) in the presence or absence of 14,15-EEZE (10 μm) for 24 h. **(A)** Chemical structures of 14,15-EET, UA-8, 14,15-EEZE. **(B)** UA-8 improved LPS-lowered cell viability. **(C)** Mitochondrial oxidative metabolic activity was preserved by treatment with UA-8. **(D)** UA-8 prevented LPS-induced increase in ADP/ATP ratio. **(E)** UA-8 attenuated LPS-impaired contractility of NCMs. Values are represented as mean ± SEM, *N* = 3–4. Significance was set at *P* < 0.05. * Significantly different from control. # Significantly different from LPS-treated cells.

In this study, NCMs were treated with LPS (1 μg/ml), a novel EET-analog, UA-8 [13-(3-propylureido)tridec-8-enoic acid (1 μM)], that possesses EET-mimetic and soluble epoxide hydrolase (sEH) inhibitory properties, and/or 14,15-EET (1 μM) as a model EETs (Batchu et al., 2011). The chemical structure and properties of the UA-8 were previously described and depicted in **Figure 1A**; UA-8 can inhibit sEH at nanomolar concentrations (IC_50_ 46 nM; Batchu et al., 2011). In order to block EET-mediated effects, we utilized the antagonist, 14,15-epoxyeicosa-5(*Z*)-enoic acid (14,15-EEZE, 10 μM). PPARγ was inhibited with a specific pharmacological agent GW9662 (1 μM; Seargent et al., 2004). Stock solutions of UA-8 and GW9662 were prepared in DMSO while 14,15-EET, and 14,15-EEZE were prepared in 100% ethanol, final concentrations of both solvents were less than 0.01% of the treatment solutions.

### METABOLIC ASSESSMENTS

In order to test overall efficiency of mitochondrial oxidative metabolism, we used a kit (Sigma–Aldrich, Co., Oakville, ON, CAN) measuring ADP/ATP ratio in cell lysates by luciferase-based method. The intensity of emitted light occurred during the first reaction is proportional to the level of ADP in the sample while the intensity of the second reaction reflects the level of ATP. Alternatively, MTT assay was employed to examine total oxidative metabolism as previously described (Samokhvalov et al., 2013). The intensity of reduction of 3-(4,5-dimethylthiazol-2-yl)-2,5-diphenyltetrazolium bromide to formazan crystals by mitochondrial dehydrogenases positively correlates with the overall activity of oxidative metabolism (Wang et al., 2010). Optical density of DMSO extracted formazan was measured spectrophotometrically at 595 nm.

### TOTAL ANTIOXIDANT CAPACITY, CASPASE-3, AND 20S PROTEASOME ACTIVITY ASSAYS

We determined the total antioxidant activity to provide an indication of the relative ROS status. Briefly, the principle of the antioxidant assay is formation of a ferryl myoglobin radical from metmyoglobin and hydrogen peroxide, which oxidizes the ABTS [2,2′-azino-bis(3-ethylbenzothiazoline-6-sulfonic acid) producing a radical cation, ABTS+, a soluble chromogen that is green in color and can be determined spectrophotometrically at 405 nm. (Sigma–Aldrich, Co, Oakville, ON, USA). To assess activation of apoptosis, we measured caspase-3 activity by employing a spectrofluorimetric assay, which detects AMC fluorescence after cleavage of AC-DEVD-AMC substrate as described previously (Seubert et al., 2002). Total proteasome activity as a marker of unspecific degenerative processes (Samokhvalov et al., 2013) was determined in the whole cell lysates based on monitoring the release of AMC by proteolytic cleavage of the peptide Suc-LLVY-AMC (CHEMICON Inc, Billerica, MA, USA) by 20S proteasomes. Fluorescence was monitored in both caspase-3 and total proteasome assays at wavelengths of 380 nm (excitation) and 460 nm (emission). Specific activities were determined from a standard curve established with AMC.

### CYTOKINES ASSAY

Medium was centrifuged (5 min at 5000 g) supernatants were analyzed by ELISA for rat TNFα and MCP-1 (ABCAM, Cambridge, UK).

### NF-kB AND PPARγ DNA BINDING ASSAYS

NF-kB DNA binding assay was measured using an ELISA kit from Active Motif (Carlsbad, CA, USA). PPARγ DNA biding activity was measured using an ELISA kit from ABCAM (Cambridge, UK). Briefly, the assays are based on the specific recognition of PPARγ or NF-kB response elements by intracellular PPARγ or NF-kB transcription factors contained in cell lysates.

### STATISTICAL ANALYSIS

Data are presented as mean ± SEM. Statistical analysis was based on one-way ANOVA with a Bonferonni *post hoc* test; *P* < 0.05 was considered statistically significant.

## RESULTS

### TREATMENT WITH UA-8 ATTENUATED LPS-INDUCED DECREASE IN CELL VIABILITY AND FUNCTIONAL ACTIVITY

Lipopolysaccharide is a well-known cytotoxic agent promoting rapid cell death (Charalambous et al., 2007). After 24 h, more than 50% of NCMs exposed to LPS were dead as evaluated by a Trypan blue exclusion assay (**Figure 1B**). Addition of UA-8 dramatically prevented LPS-triggered decrease in cell viability. However, co-treatment with 14,15-EEZE, an EET antagonist, abolished UA-8 protection against LPS (**Figure 1B**). In order to further examine protective effect of UA-8, we examined total oxidative metabolism in cardiomyocytes exposed to LPS by MTT assay. **Figure 1C** demonstrates that treatment with LPS for 24 h caused a significant decline in oxidative metabolic activity suggesting that mitochondrial function was severely compromised. Treatment with UA-8 ameliorated LPS-induced exacerbation in mitochondrial function, which was in turn abolished by a co-treatment with 14,15-EEZE. Paralleling our observations with regard to impairments in mitochondrial function, LPS also caused a robust increase in ADP/ATP ratio that was greatly reversed by addition of UA-8 (**Figure 1D**), suggesting mitochondria in LPS treated NCMs could no longer meet cellular demands for ATP. The ability of cardiomyocytes to contract *in vitro* reflects their functional activity and requires a continuous supply of ATP to sustain normal contractility (Yasuda and Lew, 1997). We found that treatment with LPS for 24 h induced a dramatic reduction in contractile activity of NCMs indicative of severe impairments in their functional activity (**Figure 1E**). Treatment with UA-8 prevented the loss of contractile activity in NCMs, which was abolished by co-treatment with 14,15-EEZE. These data demonstrate that the LPS triggered mitochondrial dysfunction in NCMs led to ATP deprivation associated with decreased contractility and resulting in cell death, was rescued by treatment with UA-8.

### LPS-INDUCED ACTIVATION OF INFLAMMATORY RESPONSE IS ATTENUATED BY UA-8

**FIGURE 2:**
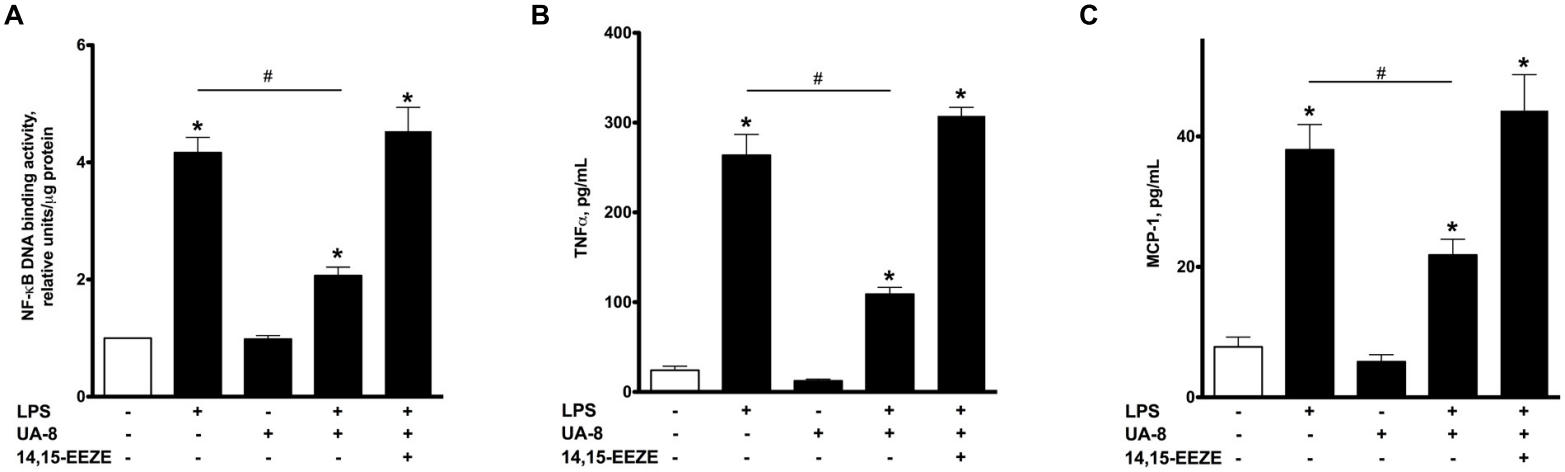
**UA-8 inhibited inflammatory response triggered by LPS in NCMs.** NCMs were treated with LPS (1 μg/ml) and/or UA-8 (1 μm) in the presence or absence of 14,15-EEZE (10 μm) for 24 h. **(A)** UA-8 suppressed LPS-triggered up-regulation of NF-kB DNA binding activity in NCMs. **(B)** and **(C)** UA-8 robustly diminished the release of TNFα and MCP-1 from NCMs exposed to LPS. Values are represented as mean ± SEM, *N* = 3–4. Significance was set at *P* < 0.05. * Significantly different from control. # Significantly different from LPS-treated cells.

Lipopolysaccharide-induced cytotoxicity is largely mediated through robust activation of the pro-inflammatory response (Charalambous et al., 2007). Therefore, it was important to investigate if inflammatory markers were up-regulated in our experimental model of LPS-induced cytotoxicity and whether treatment with UA-8 could reduce them. Accordingly, we first assessed NF-kB DNA binding activity, a test revealing functional activity of the major factor orchestrating the pro-inflammatory response. **Figure 2A** illustrates that treatment with LPS for 24 h induced a pronounced increase in NF-kB DNA binding activity. Furthermore, we also demonstrate that LPS promoted a strong release of the major pro-inflammatory cytokines TNFα and MCP-1 from cardiomyocytes (**Figures 2B,C**). These changes in the levels of the inflammatory markers indicate a profound activation of the pro-inflammatory response caused by LPS. Co-treatment of cells with UA-8 significantly attenuated the LPS-triggered inflammatory response in cardiomyocytes as seen with reduced NF-kB DNA binding activity and decreased release of TNFα and MCP-1 cytokines. Co-treatment with 14,15-EEZE abolished the anti-inflammatory effects of UA-8, thus suggesting the UA-8 associated improvements in LPS-triggered inflammatory response were realized through EET-specific pathways.

### UA-8 AMELIORATES CELLULAR STRESS REACTIONS IN RESPONSE TO LPS

**FIGURE 3:**
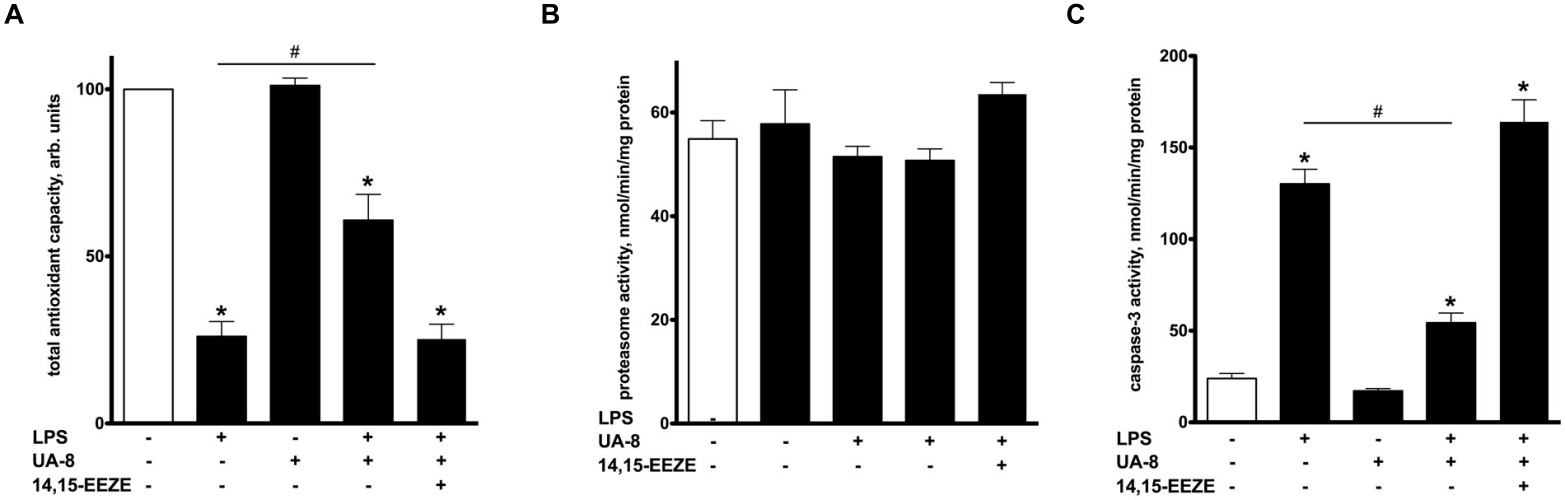
**Treatment with UA-8 reduced LPS-evoked activation of cellular stress responses.** NCMs were treated with LPS (1 μg/ml) and/or UA-8 (1 μm) in the presence or absence of 14,15-EEZE (10 μm) for 24 h. **(A)** UA-8 sustained the total antioxidant capacity of NCMs exposed to LPS. **(B)** No changes were observed in 20S total proteasome activity after treatment either with UA-8 or with LPS. **(C)** UA-8 prevented LPS-induced activation of caspase-3. Values are represented as mean ± SEM, *N* = 3–4. Significance was set at *P* < 0.05. * Significantly different from control. # Significantly different from LPS-treated cells.

Oxidative stress has been recognized as a major unspecific stress reaction mediating LPS cytotoxicity (Ben-Shaul et al., 2001). In order to examine the involvement of oxidative stress in LPS-induced cytotoxicity, we employed a test measuring a total pool of enzymatic and non-enzymatic components of the cellular antioxidant defense, thereby revealing cell ability to withstand oxidative stress. We found that LPS caused a collapse in total antioxidant capacity of cardiomyocytes indicative of activated oxidative stress. However, treatment with UA-8 significantly preserved the total antioxidant capacity of LPS-exposed cardiomyocytes, thus, providing a piece of evidence that activation of oxidative stress did not occur in full (**Figure 3A**). The accumulation of ubiquinated proteins triggers 20S proteasome activity to remove the targeted damaged proteins. As such, 20S proteasome activity can be utilized as a marker of unspecific cellular degenerative processes (Samokhvalov et al., 2013). Interestingly, treatment with LPS failed to induce any alterations in 20S proteasome activity (**Figure 3B**). LPS triggers a number of complex degenerative reactions culminating in cell death, often through apoptosis (Turdi et al., 2012). Consistent with these reports, **Figure 3C** demonstrates that treatment with LPS provoked a robust activation of caspase-3, which indicates initiation of apoptosis. Treatment with UA-8 dramatically decreased LPS-induced activation of caspase-3. Importantly, all observed effects of UA-8 were abolished by a co-treatment with 14,15-EEZE providing further support for the involvement of specific EET’s signaling.

### PPARγ INHIBITION PREVENTS UA-8-ASSOCIATED PROTECTIVE EFFECTS IN LPS-INDUCED CYTOTOXICITY

**FIGURE 4:**
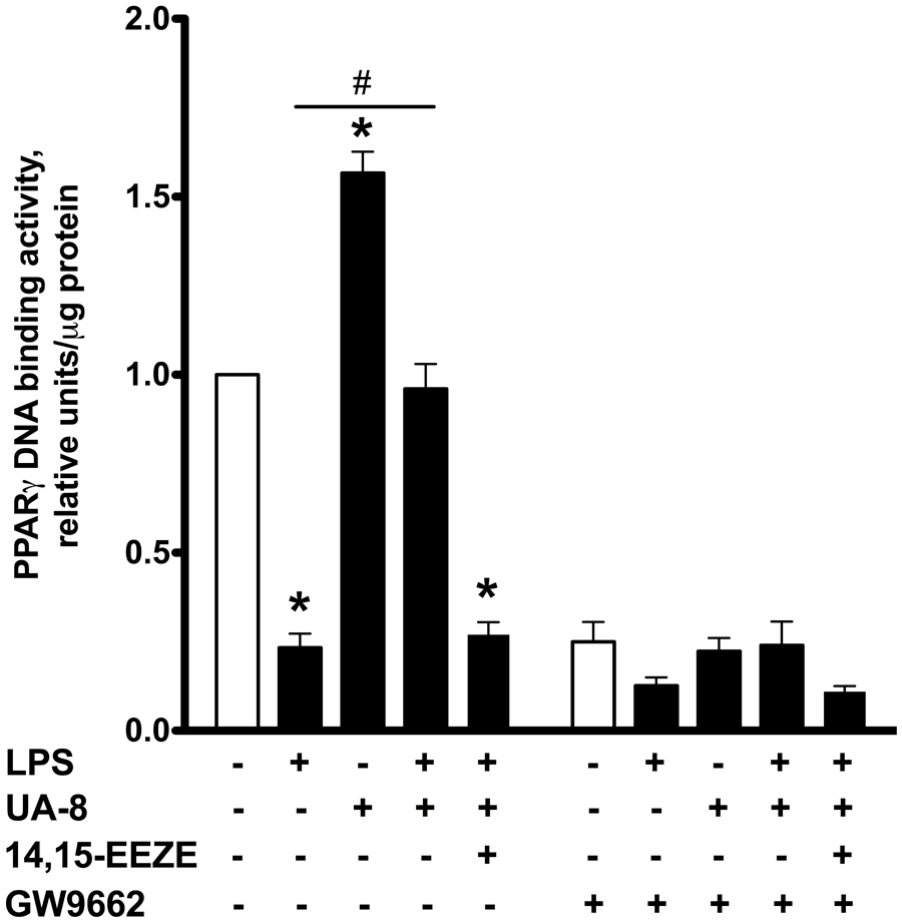
**Effect of UA-8 and LPS treatments on PPARγ DNA binding activity in NCMS and its pharmacological inhibition with GW 9662.** NCMs were treated with LPS (1 μg/ml) and/or UA-8 (1 μm) in the presence or absence of 14,15-EEZE (10 μm) for 24 h. GW 9662 (1 μm), a pharmacological inhibitor of PPARγ, was added when indicated. Values are represented as mean ± SEM, *N* = 3–4. Significance was set at *P* < 0.05. * Significantly different from control. # Significantly different from LPS-treated cells.

**FIGURE 5:**
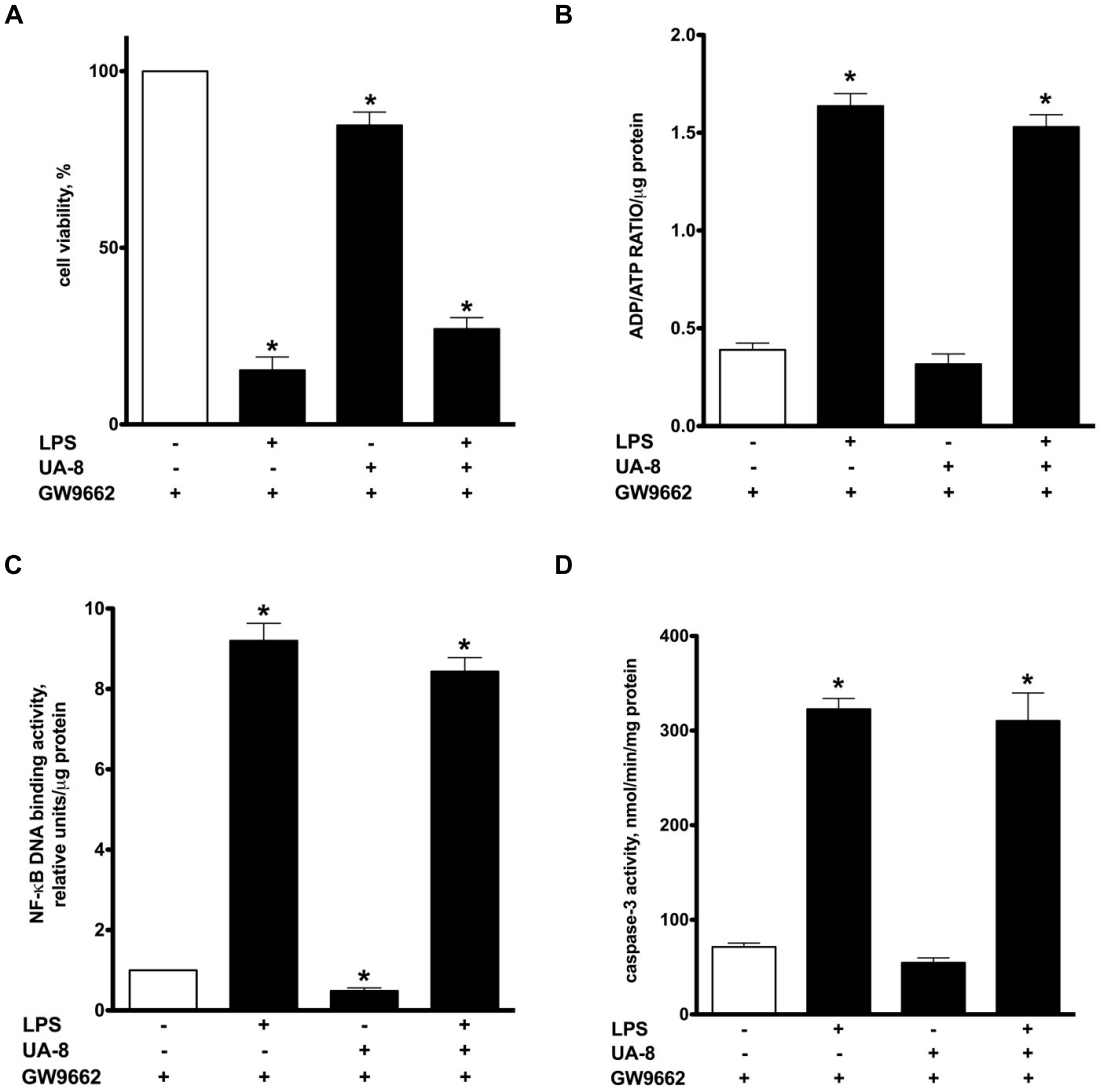
**Pharmacological inhibition of PPARγ significantly prevented UA-8 associated protective effects in LPS-induced cytotoxicity.** NCMs were treated with LPS (1μg/ml) and/or UA-8 (1 μm) in the presence of GW 9662 (1 μm) for 24 h. **(A)** Pharmacological inhibition of PPARγ prevented protective effect of UA-8 on cell viability. **(B)** Inhibition of PPARγ abolished protective effect of UA-8 on ADP/ATP ratio in NCMS exposed to LPS. **(C)** UA-8 did not reduce LPS-enhanced NF-kB DNA binding activity in NCMs treated with the pharmacological inhibitor of PPARγ. **(D)** Inhibition of PPARγ limited UA-8 inhibitory effect on caspase-3 activity in NCMs exposed to LPS. Values are represented as mean ± SEM, *N* = 3–4. Significance was set at *P* < 0.05. * Significantly different from control.

Peroxisome proliferator-activated receptors nuclear receptors are broadly recognized as signaling factors, which are involved in regulating inflammatory responses. Activation of PPARs can both stimulate an anti-inflammatory response and suppress the pro-inflammatory response. While EETs activate PPARs through ligand-specific interaction (Liu et al., 2005; Ng et al., 2007), LPS causes a reduction in the expression of PPARs (Feingold et al., 2004; Maitra et al., 2009). Thus, we explored whether UA-8 associated protective effects occurred through PPAR-signaling. First, we assessed if treatments with UA-8 and/or LPS could affect PPARγ DNA binding activity. We found that treatment with LPS caused a dramatic reduction in PPARγ DNA binding activity. In contrast, treatment with UA-8 strongly enhanced PPARγ DNA binding activity. Furthermore, treatment with UA-8 also significantly restored LPS-induced drop in PPARγ DNA binding activity (**Figure 4**). In order to examine the role of PPARγ, we treated cardiomyocytes with GW 9662, a specific pharmacological inhibitor of PPARγ signaling. Pharmacological inhibition of PPARγ with GW 9662 (1 μM) was confirmed by assessing DNA binding activity, which showed strongly reduced PPARγ DNA binding in all experimental groups (**Figure 4**). Pharmacological inhibition of PPARγ prevented UA-8-associated protective effects. Our data demonstrate that treatment with GW 9662 blocked UA-8 improved cell viability (**Figure 5A**) and ADP/ATP ratio (**Figure 5B**) in LPS-treated NCMs. Furthermore, pharmacological inhibition of PPARγ with GW 9662 prevented UA-8-evoked anti-inflammatory and anti-apoptotic effects as observed with NF-kB DNA binding (**Figure 5C**) and caspase-3 activities (**Figure 5D**) in NCMs exposed to LPS. These results provide evidence that UA-8-associated protective effects require activation of PPARγ signaling.

### 14,15-EET RECAPITULATES PROTECTIVE EFFECTS ASSOCIATED WITH UA-8

**FIGURE 6:**
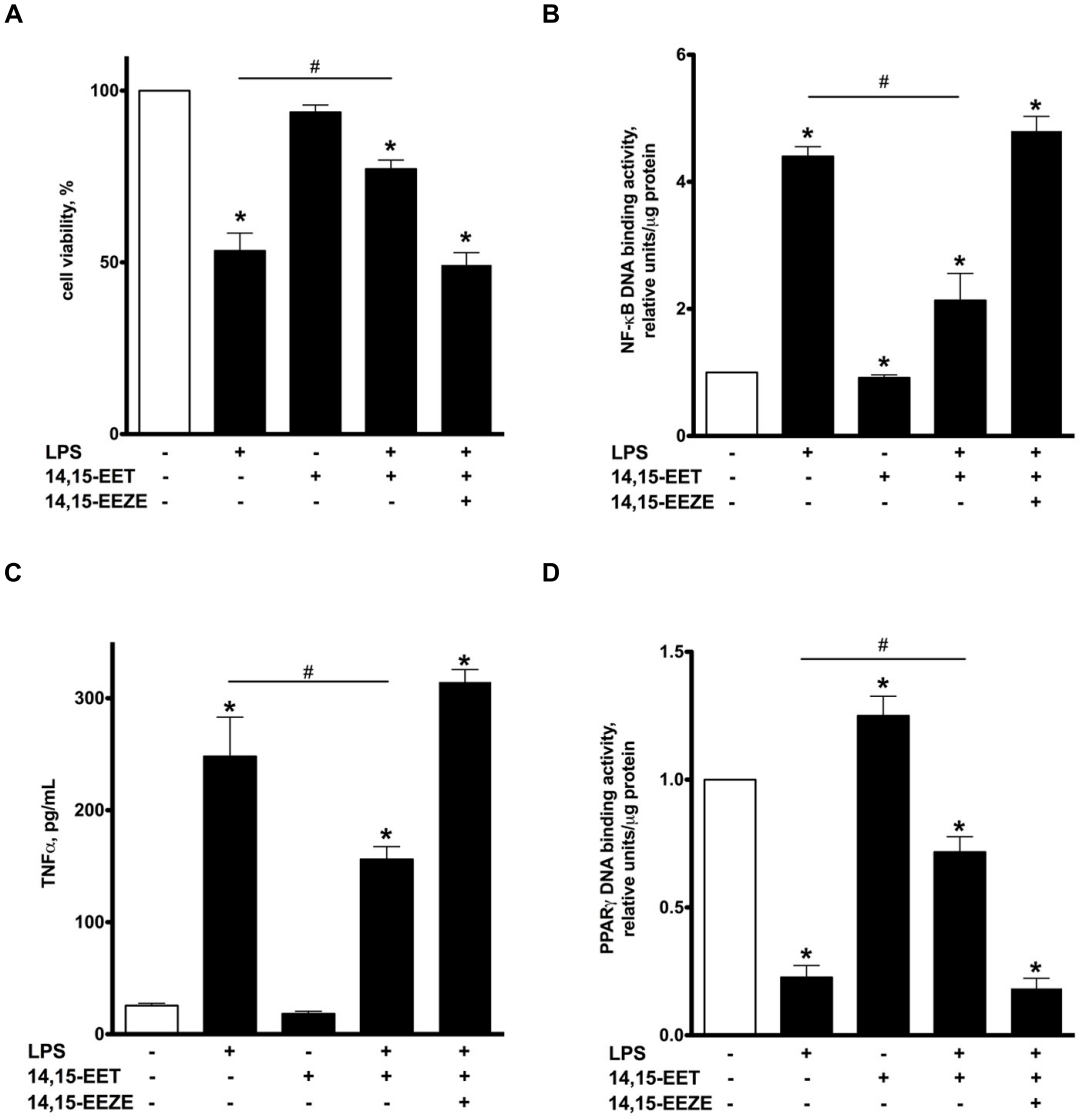
**Treatment with 14,15-epoxyeicosatrienoic acids (EET) recapitulated protective effects of UA-8 in LPS-induced cytotoxicity.** NCMs were treated with LPS (1 μg/ml) and/or 14,15-EET (1 μm) in the presence or absence of 14,15-EEZE (10 μm) for 24 h. **(A)** Treatment with 14,15-EET significantly improved LPS-decreased cell viability. **(B,C)** 14,15-EET significantly attenuated LPS-triggered inflammatory response as seen with reductions in NF-kB DNA binding activity and TNFα release. **(D)** 14,15-EET induced an increase in PPARγ DNA binding activity and significantly prevented LPS-triggered drop in PPARγ DNA binding activity. Values are represented as mean ± SEM, *N* = 3–4. Significance was set at *P* < 0.05. * Significantly different from control. # Significantly different from LPS-treated cells.

UA-8 represents a synthetic compound with structural similarities to EETs and sEH inhibitor properties (Batchu et al., 2011). Thus, in order to further determine if the effects were mediated by EET-mediated events, we utilized 14,15-EET as a model to explore similarities observed with UA-8. NCMs treated with 14,15-EET (1 μM) demonstrated significantly better cell viability following LPS treatment (**Figure 6A**). Furthermore, treatment with 14,15-EET significantly reduced LPS-triggered NF-kB DNA binding activity and release of TNFα (**Figures 6B,C**). These observations illustrate that treatment with 14,15-EET effectively suppressed LPS-induced pro-inflammatory responses in NCMs. Finally, treatment with 14,15-EET significantly enhanced PPARγ DNA binding activity while limited LPS-induced drop in PPARγ DNA binding activity in NCMs (**Figure 6D**). Importantly, addition of 14,15-EEZE abolished all protective effects of 14,15-EET similarly as it was observed with UA-8. Thus, protective effects of UA-8 and 14,15-EET in LPS-induced cytotoxicity were biologically very similar and sensitive to 14,15-EEZE suggesting they occurred through an eicosanoid-specific signaling pathway(s).

## DISCUSSION

In the present study, we provide evidence that EET-mediated activation of PPARγ signaling is required to reduce LPS-induced cytotoxicity in cardiomyocytes, demonstrating EETs may directly regulate cardiac inflammatory responses.

Myocardial exposure to LPS can trigger low-grade inflammation reactions, which are initiated through NF-κB signaling leading to release of cytokines such as TNFα and MCP-1 (Charalambous et al., 2007). The subsequent stress and inflammatory response triggers a cascade of events that may cause deleterious alterations in mitochondrial function, leading to apoptotic cell death (Oddis and Finkel, 1995; Ben-Shaul et al., 2001; Choumar et al., 2011). For example, LPS treatment has been reported to induce depletion of cardiac ATP, which was associated with myocardium dysfunction (Drosatos et al., 2011). Consistent with the literature, our data demonstrated that LPS induced a rapid loss of cell viability and reduced mitochondrial oxidative metabolism. As expected, LPS triggered a robust activation of the pro-inflammatory response in cardiomyocytes as was seen with up-regulation of NF-kB DNA binding activity and a dramatic reduction in PPARγ DNA binding activity, which were followed by the release of TNFα and MCP-1. The LPS-instigated execution of the pro-inflammatory response was potentially the primary event causing activation of caspase-3 and collapse of the antioxidant capacity in NCMs.

In this study, we demonstrate that EETs stimulate a number of adaptive responses enabling NCMs to withstand LPS-induced cytotoxicity. Particularly, our data highlight that treatment with EETs preserved cell viability, oxidative metabolic activity, diminished the pro-inflammatory response and reduced caspase-3 activity in cardiomyocytes exposed to LPS. The protective effects of EETs were abolished by a co-treatment with its specific antagonist 14,15-EEZE providing evidence that the observed effects were attributed to EETs specific pathways. There are two key results that suggest the effects observed in the current study are attributed to EETs: (1) the biological response was completely attenuated by the EET-antagonist 14,15-EEZE; and, (2) we observe similar biological effects with 14,15-EET and UA-8. Thus, we can conclude the biological effects are mostly attributed to EET-mediated effects.

Although there is no EET specific receptor discovered yet, numerous studies show the ability of EETs to act as cellular signaling molecules regulating numerous pathways (Imig, 2012; Shahabi et al., 2014). Evidence suggests that EETs may act through a receptor(s) and demonstrate affinity to known receptors (Widstrom et al., 2003; Spector and Norris, 2007). Currently, there is limited information regarding the exact concentrations of EET regioisomers found in cardiomyocytes but evidence indicates bioavailability is influenced by various factors such as stress (Imig, 2012). Intriguingly, EETs may act as endogenous agonists to PPARs, which increases VEGF and angiogenesis in endothelial progenitor cells (Liu et al., 2005; Ng et al., 2007; Xu et al., 2013). In contrast, LPS has been shown to cause a dramatic reduction in the expression of PPARs (Feingold et al., 2004; Maitra et al., 2009). Previously, we reported that treatment with LPS resulted in a rapid decrease in PPARα DNA binding activity in NCMs and PPARγ DNA binding activity in peritoneal macrophages. The primary observation from this study was that LPS-triggered a rapid decline in PPARα and PPARγ DNA binding activities evoked a robust pro-inflammatory response in both NCMs and peritoneal macrophages. Furthermore, restoring PPARα and PPARγ DNA binding activities by inhibiting malonyl-CoA decarboxylase significantly limited LPS-triggered inflammatory response (Samokhvalov et al., 2012). This may be a compelling observation validating the importance of finding novel agonists of PPARs to develop new strategies targeting LPS-induced cytotoxicity. Numerous reports postulate that activation of PPAR-signaling, particularly PPARα and PPARγ, can suppress the inflammatory response through inhibition of NF-kB pathways (Liu et al., 2005; Wray and Bishop-Bailey, 2008). Nonetheless, the protective effects of EETs are not limited to activating PPARγ-mediated signaling pathways. There is strong evidence in the literature that EETs suppress NF-kB-mediated induction and the subsequent pro-inflammatory response through inhibition of IKK complex activity (Deng et al., 2010), which partially occurs via activation of PI3K-dependent Akt and EGF receptor signaling pathway (Zhao et al., 2012). The involvement of PI3K signaling in EET-mediated activation of PPARγ is possible mechanism of action. Our previously published studies suggest that EET triggered biologically effects might involve PI3K (Batchu et al., 2011, 2012). Evidence from the literature also suggests a complex interplay between PI3K and PPARγ pathways (Chuang et al., 2007; Mishra et al., 2010). Although these indirect pieces of evidence support the role of PI3K cascade in EET-mediated events, further evidence is required to understand the precise mechanism(s) of action. Considering evidence showing desensitization to LPS is favorable in patients with chronic heart failure (Charalambous et al., 2007), our current data imply targeting PPARγ-mediated signaling with EETs would be a possible novel therapeutic approach to treat LPS-induced cardiac pathologies.

Enhanced release of pro-inflammatory cytokines from the heart causes recruitment of mononuclear cells which has been shown to have a role worsening the development of cardiovascular disease (Zernecke et al., 2008). The therapeutic potential for limiting inflammatory-induced migration of immune cells in myocardium appears to be tremendous. Our findings illustrate for the first time that EETs can effectively reduce LPS-triggered release of the pro-inflammatory cytokines from NCMs. While not definitive, we can still tentatively suggest that acting as agonists to PPARγ, EETs can suppress LPS-induced pro-inflammatory response thereby reducing cell death in NCMs. Indeed, our observations indicate that activation of PPARγ signaling contribute significantly to EETs exerted protective effect. This notion was supported when EET-associated protective effects were abolished by pharmacological inhibition of PPARγ signaling.

**FIGURE 7:**
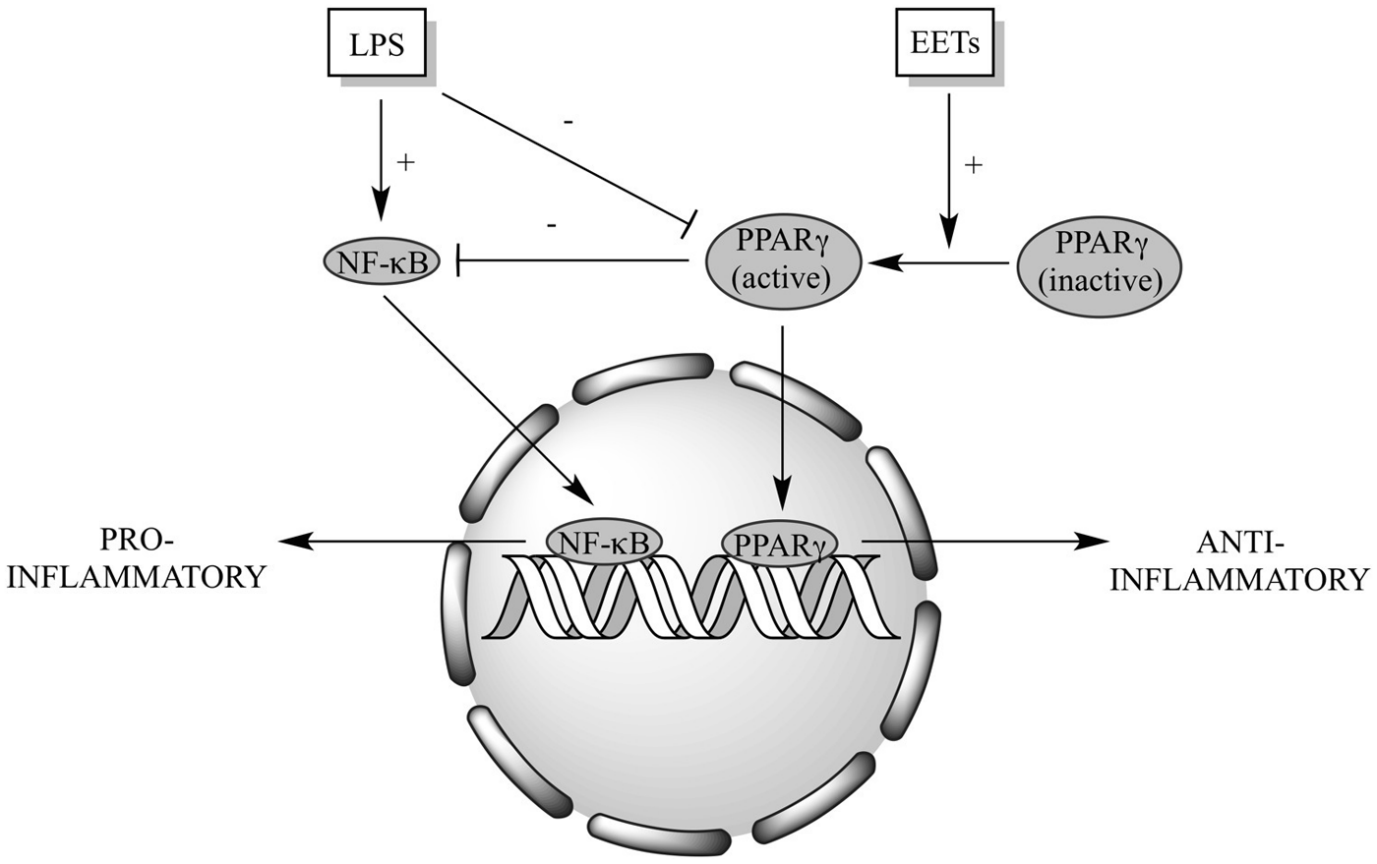
**Schematic of proposed EET cardioprotective mechanism(s).** LPS triggers activation of NF-κB-dependent signaling and suppresses PPARγ activation, resulting in a shift of the cellular response to a pro-inflammatory state. Increased EETs can act as PPARγ agonists and inhibit NF-κB-dependent signaling, shifting the cellular response to an anti-inflammatory state.

In summary, we demonstrate a crucial role of PPARγ signaling in mediating EET protective effects toward LPS-induced cytotoxicity in cardiomyocytes (**Figure 7**). While the precise cellular and molecular mechanism(s) remain unknown, our data suggest EETs exert their protective effect through activation of anti-inflammatory processes.

## Conflict of Interest Statement

The authors declare that the research was conducted in the absence of any commercial or financial relationships that could be construed as a potential conflict of interest.
